# Supplementary material for: Epitaxial nucleation and lateral growth of high-crystalline black phosphorus films on silicon
Source: Nat Commun. 2020 Mar 12;11:1330. doi: 10.1038/s41467-020-14902-z (PMC7067838; doi:10.1038/s41467-020-14902-z)
Supplement: Supplementary file 1 — Supplementary Information [file 41467_2020_14902_MOESM1_ESM.pdf]

Supplementary Information

Epitaxial nucleation and lateral growth of high-crystalline black phosphorus films on silicon

Yijun Xu et al.

Supplementary Figures

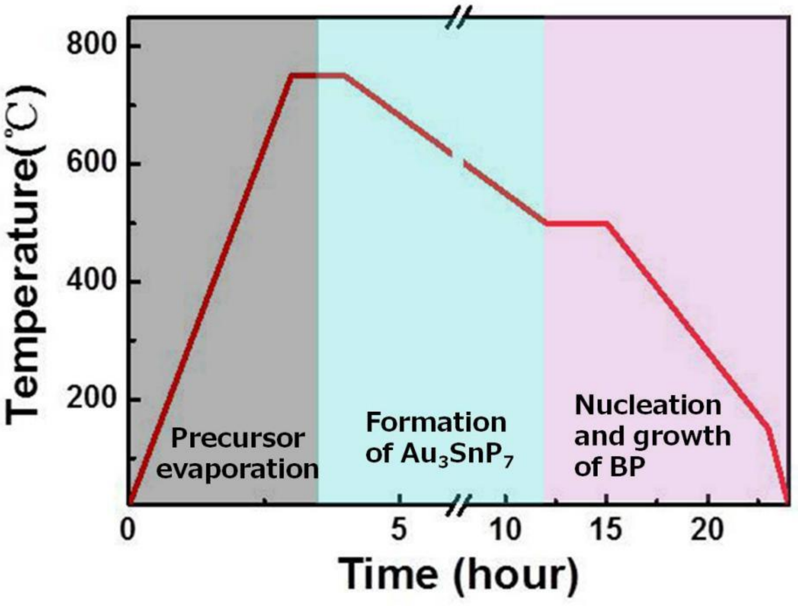

Supplementary Figure 1. The process of the nucleation and growth of BP crystalline nanofilms. The tubes, enclosed with Si/Au (thickness ≦100 nm) and precursors including Red P (RP), Sn, SnI_4, were placed horizontally in the heating zone of a quartz tube furnace. The schematic diagram for the growth process of the BP nanofilms is shown as Fig. S1 and described as following: First, the furnace was heated to 750 ℃, RP evaporated and transferred into P_4 phase, while SnI_4 decomposed to Sn and I_2. After holding at 750 ℃ for 1 hour the furnace was cooled to 500 ℃, at which stage Au film and excessive red P and Sn form into distributed Au_3SnP_7 nano-islands on silicon, which play the critical role of nucleation and growth of BP. When holding at 500 ℃, the P_4 molecules transform to six-membered P ring alone the (010) plane in Au_3SnP_7, representing the nucleation of BP. With the further decreasing of the temperature, P_4 fully condensed and transformed to BP, spatially growing along the nuclei on substrates. Distributed BP nanosheets will be gradually formed on Si substrates and finally fuse across with each other to be large BP nanofilms.

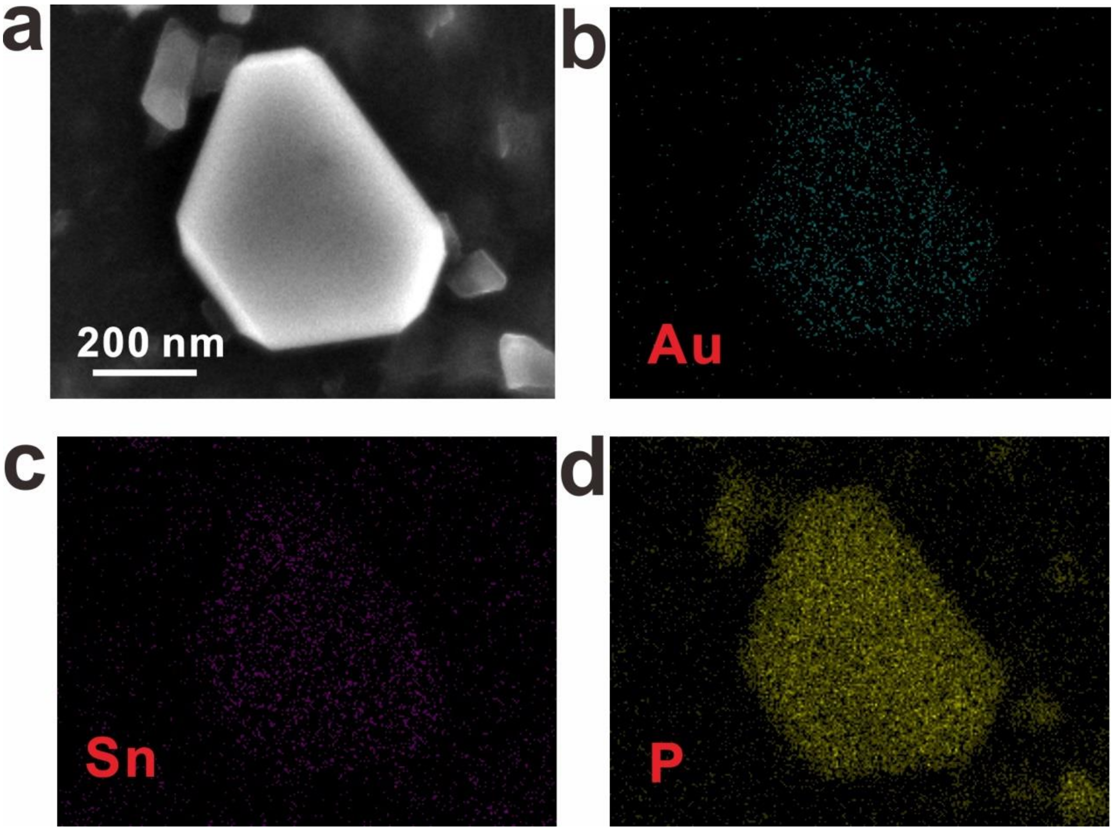

Supplementary Figure 2. Elementary analysis of the Au_3SnP_7 formed on Si/SiO_2 substrate as the nucleation seeds for the BP nanofilms growth. a, SEM image of a representative Au_3SnP_7 nano-crystal formed on Si/SiO_2 substrate. The corresponding element mapping of **b,** Au, **c,** Sn and **d,** P. Elementary analysis proves the formation of Au_3SnP_7 on the Si substrate.

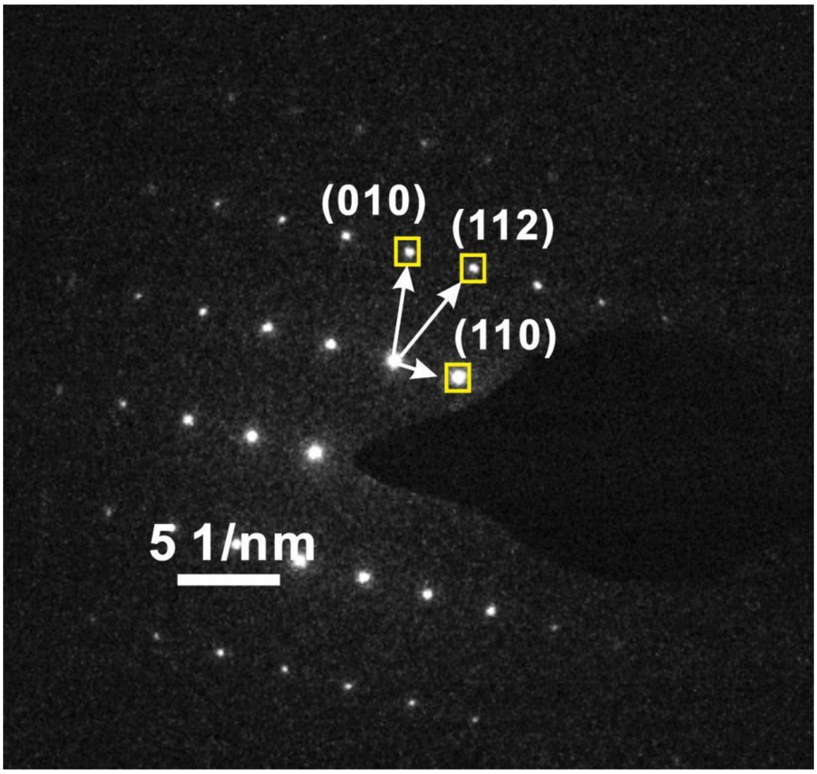

Supplementary Figure 3. The SAED proves the high crystallinity of the Au_3SnP_7 nucleation seed. The sharp and intense diffraction spots in the SAED pattern proves the high crystallinity of the Au_3SnP_7.

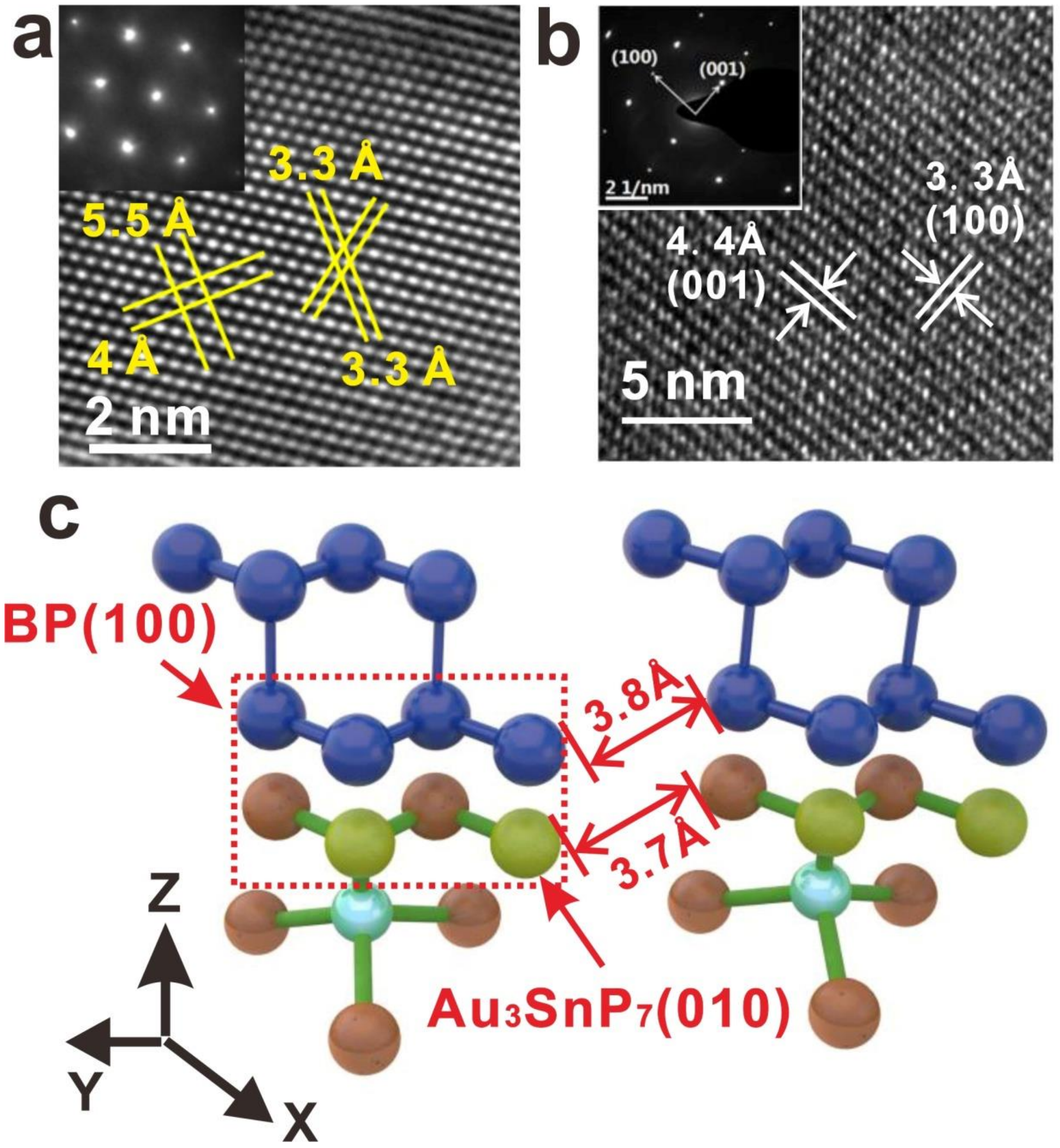

Supplementary Figure 4. A comparison of the crystal structure of the BP with Au_3SnP_7. a, HRTEM image of the Au_3SnP_7 nucleation seed grown on Si/SiO_2 substrate. Inset shows the corresponding SAED pattern of the Au_3SnP_7, indicates its single-crystalline characteristic. The lattice spacing of the Au_3SnP_7 along (010) and (110) directions are 3.3 Å and 5.5 Å, respectively. **b,** HRTEM image of the BP film grown on Au_3SnP_7 nucleation seed, inset shows the corresponding SAED pattern of the BP film. The lattice parameters of BP along (100) and (001) directions are 3.3 Å and 4.4 Å, respectively. The space group of the Au_3SnP_7 and BP is $P2_1/m$ (11) and Cmce (64), respectively. **c,** Schematic image of the crystal structure of BP (top part)

and Au_3SnP_7 (bottom part). It can be observed obviously BP along the (100) plane and Au_3SnP_7 along the (010) plane share very similar crystal structure to each other, which makes the P_4 phase prefer to transform to be BP and epitaxially nucleated on Au_3SnP_7.

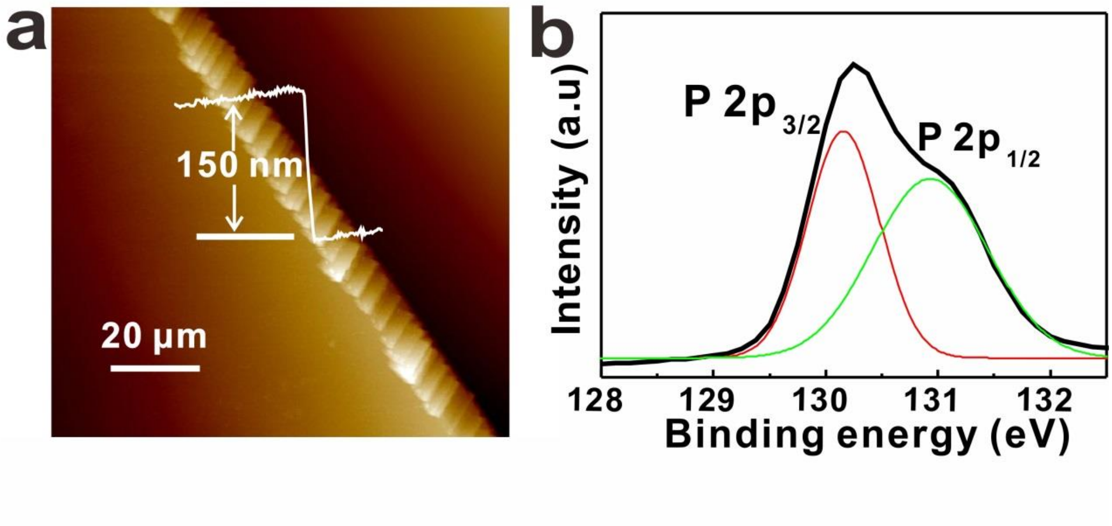

Supplementary Figure 5. Morphology and element measurement of as-grown BP films. a, AFM image of the BP film shown in fig. 1e. It exhibits a clean and flat surface with a thickness of about 150 nm. **b,** XPS spectra of the BP films grown on Si substrate, which prove its high crystallinity and purity. The XPS peak was fitted into two peaks of $2p_{3/2}$ and $2p_{1/2}$, with binding energy (BE) of 130.15 and 130.9 eV, respectively.

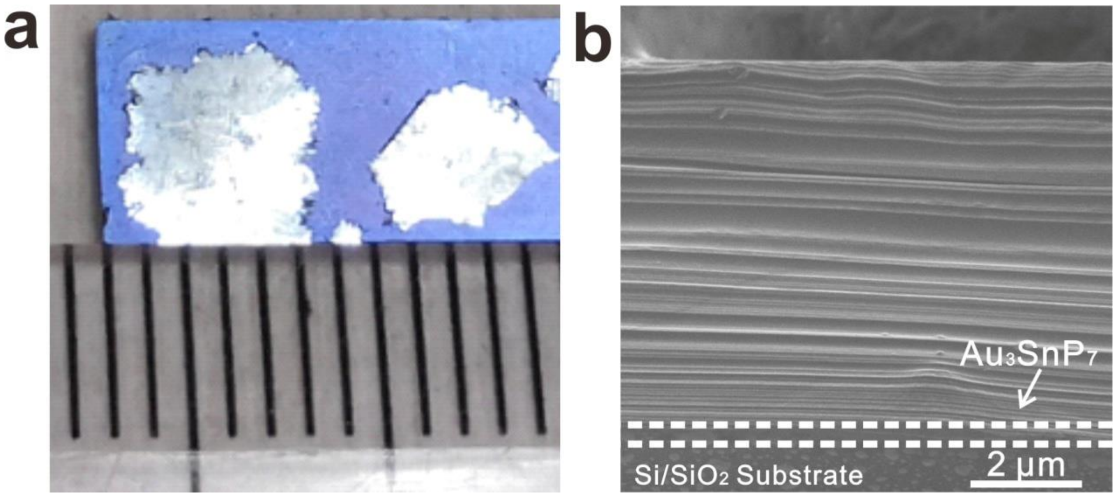

Supplementary Figure 6. The BP film can grow as large as millimeter in lateral size. a, Optic image of a typical millimeter-size BP film grown on Si/SiO_2 substrate, the BP can grown as large as 4 mm in lateral size. **b**, Cross-sectional SEM images of the as-grown BP nanofilm in **a** displays a unique lamellar structure with a thickness around 10 μm.

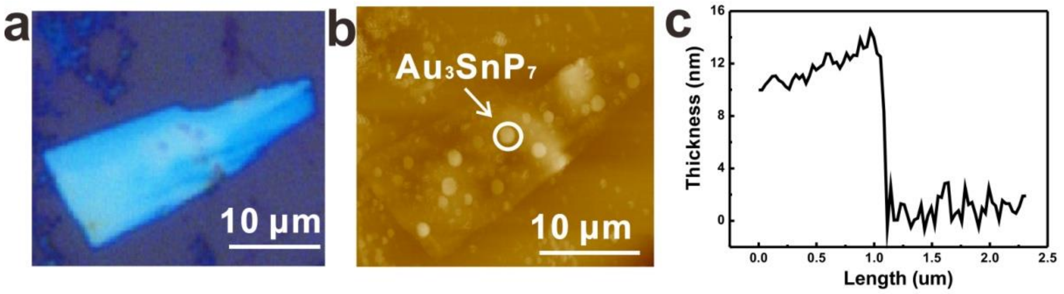

Supplementary Figure 7. Experimental proof for the growth of BP films on Au_3SnP_7 nucleation seeds. a, Optic image of a typical BP film grown on Si/SiO_2 substrate. From the top view, the surface of the BP is very smooth and clean, without any residues or adsorbate. **b,** AFM image of the BP nanofilm in **a.** Accompanied with the BP film, amount of regular shaped nano-crystals can be obviously observed, which we believe to be Au_3SnP_7 beneath the as-grown BP nanofilm. This could be another proof confirming the growth of BP nanofilm based on the Au_3SnP_7 nucleation seeds. **c,** Line-scan profile of the BP nano film shown in **a,** indicates the thickness of the BP of ~12 nm. Exist of the Au_3SnP_7 nucleation seeds can be also reflected from the fluctuations within the line scan, where the profile of the nano-islands with thickness of several nanometers are present.

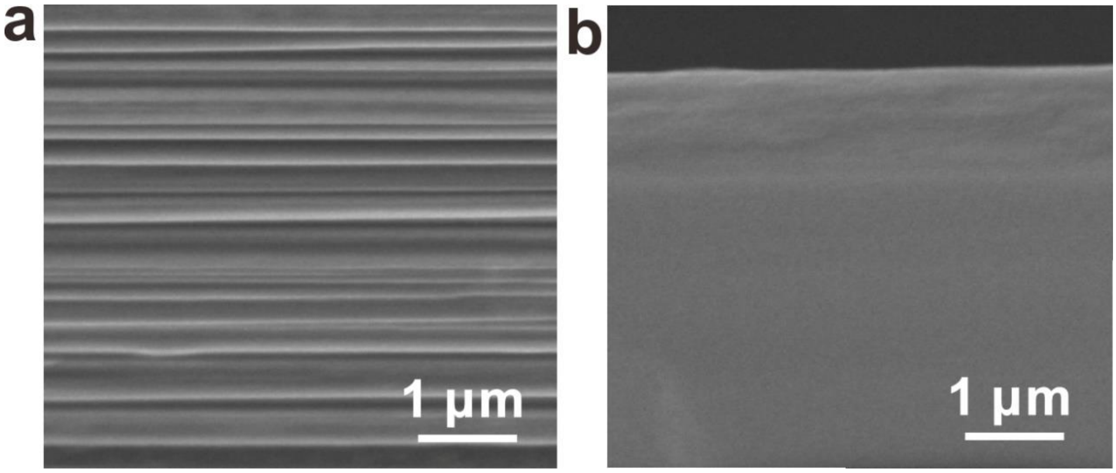

Supplementary Figure 8. A comparison of the microstructures of the lamellar structure BP film as-grown on Si/SiO_2 substrate with the conventional densely squeezed layered (DSL) BP bulk crystal. Cross-sectional SEM images of **a**, the as-grown BP film and **b**, conventional bulk BP crystals. It clearly show that our synthesized BP films own a unique lamellar structure as demonstrated in this work, quite different from the densely squeezed layered structure of the conventional BP bulk crystals synthesized before.

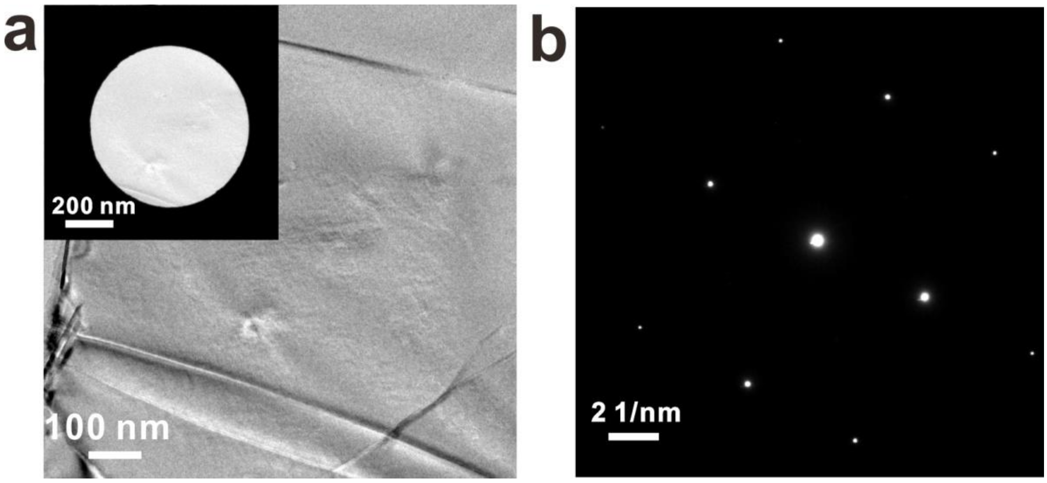

Supplementary Figure 9. Large-scale SAED pattern of the as-grown BP film. a, A typical TEM micrograph of as-grown BP film. To study the crystallinity of the BP film in larger scale, diffraction patterns were taken at different locations of the film with larger selected-area diffraction aperture of 700 nm in diameter (Fig. 9a, insert). **b,** displays a representative SAED pattern of the BP film with the larger selected-area diffraction aperture, The sharp and intense diffraction spots in the typical SAED pattern (shown in insert of Fig. 9b) proves the high crystallinity nature of the BP film over hundreds of nanometers.

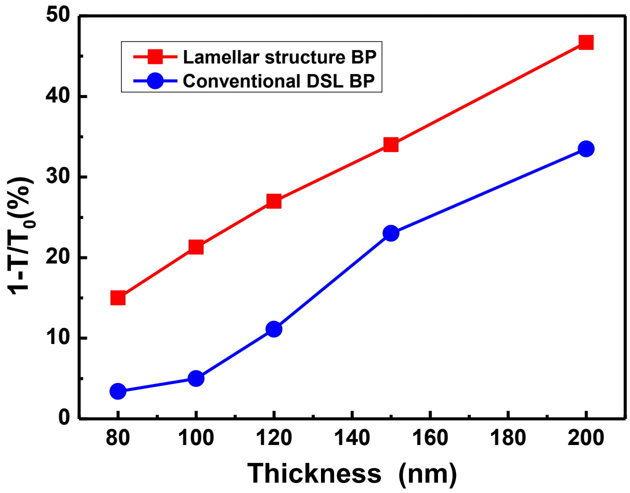

Supplementary Figure 10. Infrared absorptions of the synthesized BP film. Infrared absorptions of BP layers with different thicknesses that derived from conventional DSL BP bulk crystal (blue line) and the as-grown lamellar structure BP films (red line).

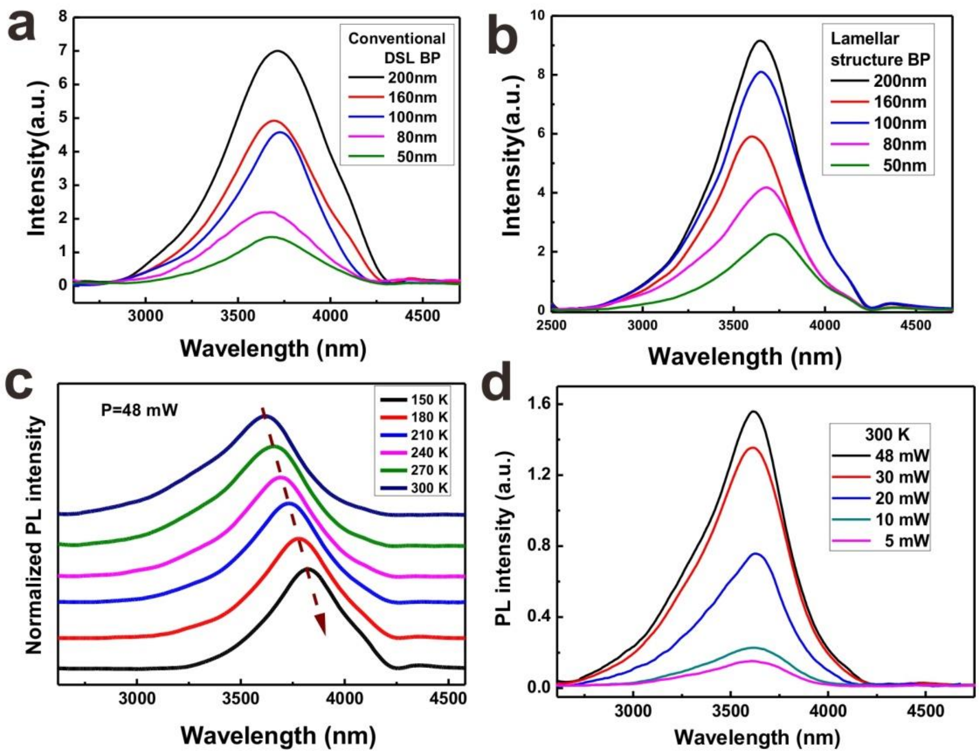

Supplementary Figure 11. Infrared photoluminescence characterizations of the synthesized BP films. a and **b**, Thickness dependent of the PL spectra of BP layers derived from conventional DSL BP bulk crystal and the lamellar structure BP films synthesized here, respectively. **c**, Temperature dependent of the PL spectra of the as-grown lamellar structure BP films. The exciton peaks displays a linear red shifts with the decreasing of temperature. **d**, Power dependent of the PL spectra of the as-grown lamellar structure BP films. All the measurements were excited by a 800 nm focused laser.

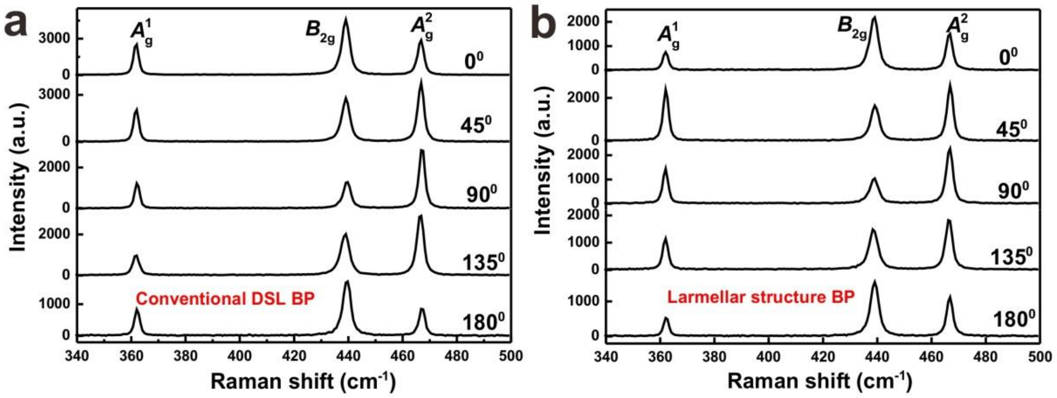

Supplementary Figure 12. Anisotropic Raman spectra of conventional DSL BP and lamellar structure BP. Raman spectra of **a**, conventional DSL BP and **b**, as-grown lamellar structure BP under different polarization angles. The incident laser was in the z direction (perpendicular to the sample), the polarization angle θ is relative to the 0° reference, which is selected at the BP armchair direction parallel to the laser polarization direction. For both conventional DSL BP and lamellar structure BP, the $A^2{}_g$ peak reaches the maximum value and the B_{2g} peak decreases to the minimum value when θ is 90°, which indicates the similar anisotropic optical properties of these two types of BP. Both conventional DSL BP and as-grown lamellar structure BP are around 10 nm in thick.

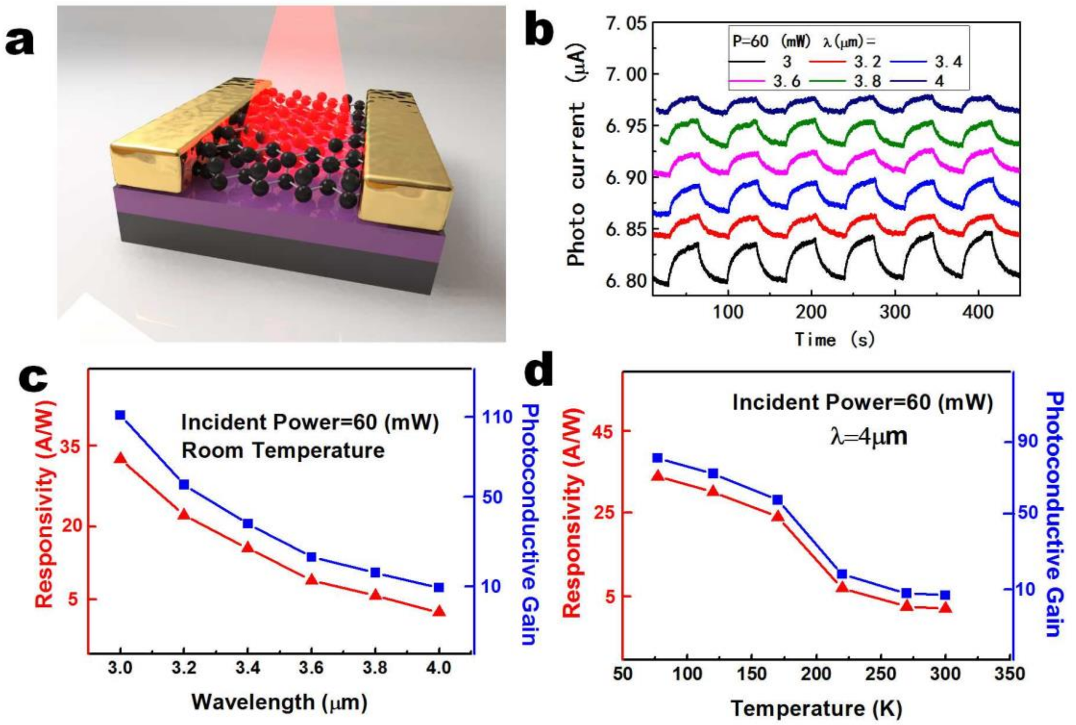

Supplementary Figure 13. The mid-infrared photodetection properties of the synthesized BP films. a, Schematic of a back-gated phototransistor employing the synthesized BP films with thickness of 12 nm. **b,** Photocurrent of the photodetector excited by the mid-infrared lasers with the laser wavelength (λ) from 3 μm to 4 μm. **c**, Photoresponsivity (R) and photoconductive gain (G) of the detectors versus detection wavelengths measured at room temperature. **d**, Temperature dependent of R and G of the BP photodetectors, measured from 300 K to 77 K with excitation laser wavelength of 4 μm. The incident power used in these measurements is 60 mW.

The detective wavelength of the BP photodetector was extended to 4 um, with R for this wavelength reaches 32 AW^{-1} at 77 K. It is worth noting that, both the photoresponsivity and photoconductive gain of the BP photodetectors demonstrated here show better performance at mid-infrared than traditional HgTe photodetector[1] and In(Ga)As/Ga(Al) quantum dot photodetector[2].

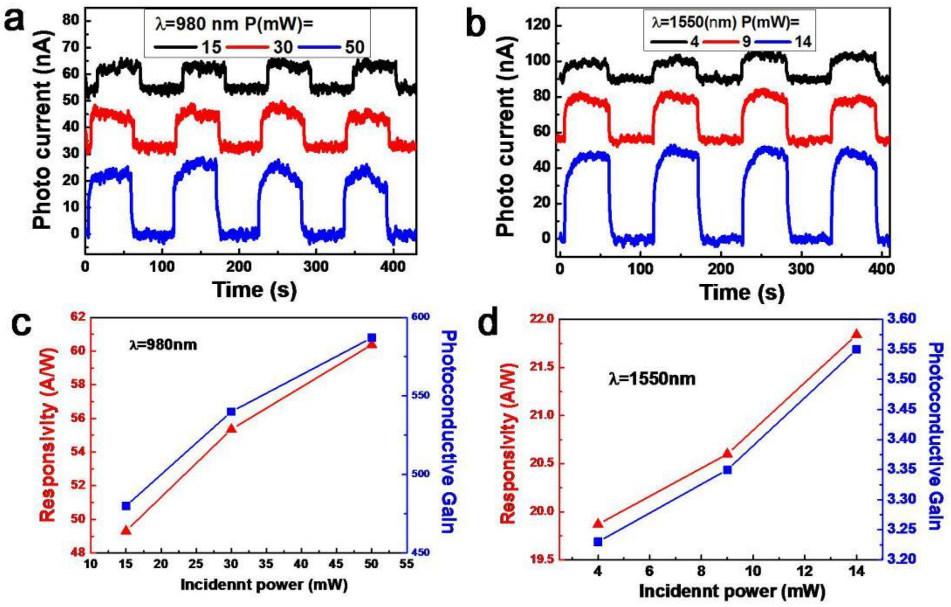

Supplementary Figure 14. The photodetection properties of the synthesized BP films at the near-infrared telecom bands. **a**, **b**, Power dependent photoresponse of the BP photodetectors excited by 980 nm and 1550 nm lasers, respectively. **c**, **d**, Photoresponsivity and photoconductive gain of the BP photodetector as a function of incident power excited by 980 nm and 1550 nm lasers, respectively. The excellent photoresponse performance measured at wavelengths of 980 nm and 1550 nm, also promises the potential use of the synthesized BP films in near-infrared telecom band communications. The BP film used for photodetectors is 12 nm in thick.

Supplementary Note 1

Calculation of carrier mobility: The field-effect carrier mobility (μ_{FE}) of the BP nanosheet can be calculated via the equation:

$$\mu_{\mathrm{FE}} = \frac{L}{WC_{\mathrm{i}}V_{\mathrm{ds}}}\frac{dI_{\mathrm{ds}}}{dV_{\mathrm{g}}} \quad (1)$$

where L and W are the length and width of the FET channel, respectively, $c_i = \varepsilon_0\varepsilon/d$ is the capacitance per unit area between BP and the silicon substrate, where $\varepsilon_0 = 8.854\times10^{-12}$ fm^{-1} is the permittivity of vacuum, ε = 3.9 is the relative dielectric constant of SiO_2, d=285 nm is the thickness of the SiO_2 layer, $\mathrm{d}I_{\mathrm{ds}}/\mathrm{d}V_{\mathrm{g}}$ represents the maximum slope extracted from the linear region of transfer characteristic.

The Hall mobility is obtained from

$$\mu_{\mathrm{H}}=\frac{L}{W}\frac{G}{ne} \quad (2)$$

Where L and W are the length and width of the channel, G is the BP sheet conductance, e is the charge of an electron and n is the carrier density.

Supplementary Note 2

Calculation of photoresponsivity and photoconductive gain: The photoresponsivity (R) and photoconductive gain (G) are two critical parameters to evaluate the photodetector performance. R is defined as the photocurrent excited by per unit power of excitation light on the effective area of a photodetector[3]:

$$R = \frac{I_{\mathrm{ph}}}{P_{\mathrm{d}}} \tag{3}$$

where $I_{\mathrm{ph}} = I_{\mathrm{laser\ on}} - I_{\mathrm{laser\ off}}$, $I_{\mathrm{laser\ on}}$ is the photocurrent exited by the incident light, I_{laseroff} is the current without illumination, P_{d} is the effective power on the device, where $P_{\mathrm{d}} = P_{\mathrm{in}}(S_{\mathrm{device}}/S_{\mathrm{spot}})$, P_{in} is the total laser power, S_{device} is the area of the device, and S_{spot} is the area of the laser spot.

Photoconductive gain is calculated by[4]:

$$G = \frac{I_{\mathrm{PH}}}{P_{\mathrm{abs}}}\frac{h\nu}{q} \tag{4}$$

where P_{abs} is the absorbed power and $h\nu$ is the photon energy. For BP thicknesses above 10 nm, the absorption coefficient α (cm^{-1}) is a constant. In this experiment, the absorption of the BP layer was calculated to be ~12% according to the Lambert−Beer's law:

$$A(\omega)=1-e^{-\alpha(\omega)\Delta z} \tag{5}$$

where $A(\omega)$ is the material absorption, ω is the light frequency, and Δz represents the thickness of the flake.

Supplementary References:

1. Keuleyan, S., Lhuillier, E., Brajuskovic,V. &Sionnest, P. G. Mid-infrared HgTe colloidal quantum dotphotodetectors. *Nature Photon.***5**, 489-493 (2011).
2. Stiff, A. D., Krishna, S. & Bhattacharya, P. High-detectivity, normal-incidence, mid-infrared (~4μm)...InAs/GaAsquantum-dot detector operating at 150 K. *Appl. Phys. Lett.* **79**, 421-423 (2001).
3. Buscema, M., Groenendijk, D. J., Blanter, S. I., Steele, G. A., van der Zant,H. S. J. & Castellanos-Gomez, A. Fast and Broadband Photoresponse of Few-Layer Black PhosphorusField-Effect Transistors. *Nano Lett.*, **14**, 3347(2014).
4. Guo, Q. S., Pospischil, A., Bhuiyan, M., Jiang, H., Tian, H., Farmer,D., Deng, B., Li, C., Han, S. J., Wang, H., Xia, Q. F., Ma, T. P.,Mueller,T. & Xia, F. N., Black Phosphorus Mid-Infrared Photodetectors with High Gain. *Nano Lett.***16,** 4648−4655 (2016).
